# Developing a Food Exchange System for Meal Planning in Vegan Children and Adolescents

**DOI:** 10.3390/nu11010043

**Published:** 2018-12-25

**Authors:** Susana Menal-Puey, Miriam Martínez-Biarge, Iva Marques-Lopes

**Affiliations:** 1 Nutrition Unit, Faculty of Health and Sport Sciences, University of Zaragoza, 22002 Huesca, Spain; smenal@unizar.es; 2 Department of Paediatrics, Imperial College London, London W12 0HS, UK; miriam.mbiarge@imperial.ac.uk

**Keywords:** food exchange, vegan diets, meal planning, vegan food portions, nutritional composition

## Abstract

Vegan diets in children need to be adequately planned so they can safely meet children’s requirements for growth and development. Adequate and realistic meal planning guidelines should not be difficult to achieve, thanks to the increasing number and availability of natural and fortified vegan foods, which can help children to meet all their nutrients requirements. In order to ensure an adequate supply of key nutrients, families and health professionals need accurate, reliable, and easy-to-use meal planning tools. The aim of this article is to provide a practical approach system to meal planning, based on the same food exchange methodology that has been already published in adults. Daily portions of each food exchange group have been calculated so the resulting menu provides at least 90% of the Dietary Reference Intakes (DRIs) of protein, iron, zinc, calcium, and n-3 fatty acids for each age group, sex, and physical activity level. These diets do not provide enough vitamin B-12 and vitamin D. Although fortified plant drinks, breakfast cereals or plant protein-rich products could provide variable amounts of these two vitamins, B12 supplementation is always recommended and vitamin D supplementation should be considered whenever sun exposure is limited. This tool can be used to plan healthful and balanced vegan diets for children and adolescents.

## 1. Introduction

The number of people consuming vegan diets is on the rise, especially in Europe and other Western countries [1,2]. Although representative data are scarce, it can be assumed that the number of infants, children, and adolescents on vegan diets is also rising [3]. Children diets are usually planned by their parents; therefore, parents' eating patterns have a large influence on children [4]. Although the American Academy of Nutrition, as well as other medical and dietetic societies, consider that well-planned vegan diets are adequate for children of all ages [5,6], a poorly planned vegan diet may have negative consequences on the health and growth of children and adolescents, similarly to any other poorly planned dietary pattern. Since energy and nutrient requirements are higher in relation to body weight during growth, infants, children, and adolescents are particularly vulnerable and are at higher risk of nutrient inadequacies than adults [3,7]. In general, studies on vegetarian and vegan adults have shown multiple health benefits, including lower risk of obesity, cardiovascular diseases, and diabetes [8,9]. Some of these effects have also been observed in children [10]. However, due to the restricted food selection, vegetarians and in particular vegans need to pay special attention to potential critical nutrients, i.e., protein, iron, zinc, calcium, vitamin D, vitamin B12, and n-3 fatty acids [11,12,13,14,15,16]. Vegan diets include only plant foods—grains, vegetables, fruits, legumes, nuts, seeds, and vegetable fats and oils. Although the likelihood of nutritional deficiencies increases with more restrictive diets, vegan diets can be adequate if parents make the appropriate food choices and follow proper meal-planning guidelines [6,7]. Health professionals and practitioners who work with vegan families need to be familiar with the nutritional issues that are specific to vegan diets and must provide adequate and realistic meal-planning guidelines. The wider availability of convenient vegan foods, many of which are fortified, is facilitating the process of planning healthful vegan diets for children. Food exchange lists have become a useful tool to monitor the intake of macronutrients in both healthy individuals and in patients with several medical conditions. We have previously developed food exchange lists using statistical criteria [17,18] that were specifically designed for vegetarian and vegan adults, and that take into account the macro- and micronutrient content of the foods typically consumed by this population [19]. Although there is scientific evidence showing that in adults a diet providing adequate amounts of energy and macronutrients would provide sufficient amounts of all minerals and vitamins [20], there are some periods during the lifecycle when micronutrient intake could be compromised despite adequate energy provision, such as childhood and adolescence. Because of the role of micronutrients in children growth and development and in the prevention of chronic diseases, it is important to develop tools that assess not only the provision of energy and macronutrients in this vulnerable population, but also the provision of key micronutrients. 

The aim of this article is to present a practical tool designed to facilitate meal planning in vegan children and adolescents. This tool has been developed using the same methodology we previously used in vegetarian and vegan adults [19]. The system is based on exchange foods, allowing the personalization of diet plans according to individual preferences, but ensuring that the resulting menu will provide sufficient macro- and micronutrients irrespective of the age, sex, and level of physical activity of the child.

## 2. Materials and Methods 

We developed this practical tool in three phases: in the first phase we decided which nutrients were of special interest in paediatric vegan nutrition, based on previous studies [11,12,13,14,15,16,21,22,23,24]. Then we selected those foods that could provide these nutrients, including several foods of each representative item (e.g., different breads, plant drinks, etc) and listed the nutritional composition of the portions usually consumed by children and adolescents. In the second phase we designed the food exchange groups, following the same methodology we have previously published; we also described the mean nutrient values of each exchange group. In the third phase we determined the number of daily portions of each exchange food group that is required to meet the nutritional and energy requirements of each age group, as explained below.

### 2.1. Phase 1

After reviewing the studies performed in vegan children over the last years, we selected the following key nutrients: vitamin B-12 and D [11,12], omega-3 fatty acids [13], protein [12,15,16], calcium, iron, and zinc [14,16,21,22,23,24]. Although a vegan diet can meet current recommendations for most of these nutrients, vitamins B12 and D can only be obtained from fortified foods, UV-irradiated mushrooms (vitamin D), or supplements (in certain circumstances vitamin D can be obtained from sunlight). Also, the use of fortified foods may be useful to ensure nutritional adequacy [16,25,26]. A vegan diet can easily meet human dietary protein requirements if the energy intake is adequate and if a variety of plant foods such as legumes, whole grains, nuts and seeds are regularly consumed; in addition soy products and meat analogues can be a valuable source of protein and minerals [6,26,27]. Vegan children can obtain calcium from some vegetables (especially cruciferous vegetables), nuts, seeds, legumes (including calcium-set tofu), and drinking water, but fortified foods such as plant drinks and some fruit juices and breakfast cereals may provide additional, easily absorbable calcium [19,26,28]. Vegans need dietary strategies to enhance the content and bioavailability of zinc and iron, since the bioavailability of these minerals from plants is lower than from animal products [16,29,30,31]. Good sources of n-3 fatty acids (alpha-linolenic acid) are ground flaxseeds, chia seeds, flaxseed oil, walnuts, and canola oil [32,33]. All vegans need to identify reliable sources of vitamin B-12 and include them in their diets. These can be fortified foods such as plant drinks, breakfast cereals, meat analogues or nutritional yeast; otherwise, the use of supplements is essential [6,16]. Vitamin D can also be obtained from fortified foods or UV-irradiated mushrooms where available [16], but in the absence of an adequate sun exposure, supplements should also be considered [3,4]. Finally, very recent studies show that attention should be paid to iodine intake, since plant-based diets can be low in iodine [15,34]. 

In order to select those foods that could provide the nutrients of interest, and list the nutritional composition of the portions usually consumed by children and adolescents, we used available information about specific foods and their macro- and micronutrient content by portion serving size [25]. We adapted portion sizes from a previously developed semiquantitative food frequency questionnaire for vegans and vegetarians in which portions of foods typically consumed by this population had been included [35]. Nutrient values of each selected food were obtained from the Spanish Food Composition Database [36] and, when necessary, from other databases, such as the United States Nutrient Database [37] or the Finnish Food Composition Database [38]. A food portion composition database was created. Food groups were selected according to their nutritional profile. Foods were classified into groups according to the predominant nutrient: (1) grains, potatoes, and legumes as foods containing primarily carbohydrates; (2) Plant protein-rich products as the foods providing mainly protein; (3) oils, nuts and seeds as foods providing mainly lipids; (4) Soy products, fortified plant drinks and other calcium-rich products; (5) fruits and vegetables as important sources of vitamins and minerals; and (6) Occasional foods as sugars and baked products.

### 2.2. Phase 2 

Foods were placed in the different food exchange lists according to their nutritional composition. All the amounts were defined in grams, and were converted into household measures and portion sizes. After definition of the food groups, the amount of each food that could be exchanged with any other food in the same group, based on their similar nutritional value, was determined. In this regard, different amounts of each food were introduced in the calibration software [38] and main macronutrient values of each one were compared, so the most appropriate amounts for matching lists could be selected. Following a previously published methodology for designing food exchange lists [17], we developed food exchange groups by calculating the mean, standard deviation (SD) and coefficient of variation (CV) values for energy, macronutrients (protein, carbohydrate and fat), and micronutrients (calcium, iron, zinc, vitamin B12 and vitamin D). To validate foods within a list, we considered the recommended values of SD used by Wheeler and colleagues [39] in exchange lists for energy and macronutrients (energy: 20 kcal, carbohydrates: 5 g, fat: 2 g, and protein: 3 g). If SD values were outside the limits, amounts of foods with greater SD were modified or the food was removed from that list and appropriately placed in another list so that it would fit its macronutrient content within that list. Once the SD was adjusted, the CV was also analysed, aiming for values less than 30%; and macronutrients and energy contents were matched. A CV of less than 30% was sought for energy and at least two macronutrients. For groups with higher values of CV, the Z value for each food was calculated. We established as criterion Z values between −2 and +2, in order to exclude foods with high variations [17]. The energy value of each exchange group was calculated by multiplying the content of protein, fats and carbohydrates assigned to each exchange list by the Atwater factors. The main criteria for defining food exchange lists were energy and macronutrients content, but the statistical values of micronutrients were also included so the contribution of each group and subgroup to the resulting menu could be monitored. In all cases, the amounts in grams tested were established based on common culinary measures, to facilitate dietetic practices (e.g., 10 g oil, 2 teaspoons) or on usual portion sizes (e.g., plant drink, 1 cup 200 mL) or on small amounts deliberately established so they could be easily converted into smaller or larger food portions if necessary (e.g., textured soy protein, 30 g, 3 tablespoons).

### 2.3. Phase 3

Three age groups were defined according to their nutritional needs (1–3 years, 4–10 years, and 11–17 years). The mean value of Dietary Reference Intakes (DRIs) for boys and girls was calculated according to the Dietary Reference Values for nutrients from the European Food Safety Authority (EFSA) summary report [40]. As energy needs differ depending not only on age and sex, but also on the level of physical activity (from sedentary to moderately active), a range of energy requirements was considered (from 900 to 3000 kcal/day). Reference values of body weight and physical activity levels from 1 to 17 years were extracted from the EFSA summary report [40].

The number of portions of each food exchange group was defined so that the resulting menu provided intakes of at least 90% of the European Dietary Reference intakes (DRI) [40] for energy, protein, n-3 and n-6 fatty acids, calcium, iron, zinc, and vitamins D and B_12_. The desired percentage of energy provided by carbohydrates and fats was established as 50–55 and 30–35 percent of total daily energy respectively. For the analysis of protein, both the requirements per kg of body weight [40] and the desired 10–15% of the total energy provided by this nutrient were taken into account. The desired percentage of energy provided by protein corresponded with an amount in total grams that was higher than the amount derived from the DRI, and that was the number selected for the calculations. 

## 3. Results

The macronutrient (protein, carbohydrate, fat) and micronutrient (calcium, iron, zinc, vitamin B12, vitamin D, n-3 fatty acids) values (mean, SD and CV values) assigned to each food list, after subjecting them to the rounding criteria and the energy value calculated by the Atwater system, are shown in Table 1. After calculating the content of other nutrients in the portions tested, the main groups were divided into subgroups, according to the content of other nutrients. Grains and tubers are the first group. The portions of grains, including those commonly consumed by vegans (millet, spelt, barley, rye, quinoa, bulgur, kamut) were adjusted (60 g bread, 40 g dry or raw cereals, 200 g potato) in order to keep the SD values of the whole group constant. Commonly consumed legumes (beans, lentils, and chickpeas) as well as other types of legumes such as soy beans, adzuki, or mungo beans and lupins were also included in this group, because they contribute carbohydrates to the diet (13.0 (2.2) g). However, they also provide protein (6.9 (0.9) g) and therefore they are included in a specific subgroup. Protein was the main macronutrient in the plant protein-rich foods group (12.9 (1.9) g), which includes tempeh, seitan, textured soy protein, and vegan burgers and similar meat substitutes; they also contribute calcium (60.3 (17) mg), and iron (2.5 (1.4) mg). To be considered in this group vegan meat-alternatives had to be made with legumes, tofu, tempeh, seitan, textured soy protein, or a mixture of these ingredients, and had to provide at least 10grams of protein per portion. Some vegan meat-alternatives are fortified with vitamin B12 and may be a source of this nutrient. Seeds usually consumed by vegans (flax, sesame, chia, sunflower, and pumpkin seeds) and nuts (almonds, cashews, hazelnuts, pistachios, pine nuts, walnuts) are a source of protein, but when nutrient contents by portions (20 g) were considered, the protein value was low (3.6 (1.3) g) and therefore insufficient for nuts and seeds to be included in the plant protein-rich group. Another important group in the vegan food pattern is the vegetable fat-rich group as they provide energy, essential fatty acids and other micronutrients. Different vegetable oils, oily fruits, nuts, seeds, and spreads commonly consumed by vegans were included. Polyunsaturated fatty acids were the main constituents, either as n-3 fatty acids in flaxseed and flaxseed oil, and/or as omega-6 in canola, soy, sunflower, walnut, sesame, and pumpkin seed oils. Based on the nutritional analysis performed on this group, and taking into account easily household measures for exchange portions, three subgroups were created: (2) Oils and oily fruits, (3) nuts, seeds, and their butters (tahini, peanut, and almond paste), and (4) n-3 fatty acids sources, including walnut, flax and chia seeds, and walnut and flaxseed oil. In vegan diets, several calcium-rich foods are proposed as a substitute for milk and dairy. After nutritional analysis, they were subdivided into (1) soy products and (2) fortified plant drinks, because of their different protein content. The soy products group included fortified soy drink, soy yogurt, and tofu. Per portion, they were good sources (mean (SD) of calcium (136.6 (52.5) mg) and protein (5.7 (1.2) g).

Fortified plant drinks other than soy (oat drink, rice drink, and almond drink) were considerably higher in calcium (284 (79.7) mg) but lower in protein (1.6 (1.2) g) as compared with soy products (mean (SD) per portion. Also, they provided significant amounts of vitamin B-12 (1.5 (0.9) mg) and D (1.8 (0.3) mg), as most brands are fortified with these nutrients. 

Within the vegetable food group, we distinguished cruciferous from other vegetables, because of their calcium contribution (mean (SD) per portion (139.5 (36) g), which is comparable to soy products (136.6 (52.5) mg). This subgroup includes broccoli, cauliflower, cabbage, kale and Brussels sprouts. Another subgroup containing sprouted vegetable portions (30 g) was included. In order to match food groups and achieve a nutritional uniformity, portions of the most commonly consumed vegetables (chard, spinach, lettuce, endive, borage, green bean, pea, eggplant, zucchini, cucumber, tomato, onion, pumpkin, carrot, pea, asparagus, artichoke, cardoon, mushrooms) were defined on the basis of a previously published Spanish food exchange list (100 to 200 g) [17].

The fruit group includes typically whole fresh fruit. Similarly to the vegetables group, different amounts of portions of fruits (orange, grapefruit, tangerine, nectarine, banana, apple, pear, blueberry blackberry raspberry, acerola, strawberry, cherry, plum, peach, apricot, watermelon, melon, pineapple, fig, pomegranate, mango, kiwi, jujube, and grape) were considered (100–250 g) [17], and dried fruits (30 g) were included (raisin, dried plum, dried fig, date, and dried apricot/ peach). These portions contributed a mean sugar content of 15.1 g (2.5 g).

We added a group containing the most typical sweeteners and baked products, because they are consumed by many children and adolescents. Nevertheless, we did not count them towards the total caloric and nutritional requirements. Portions of the sugars group (white and brown sugar, soluble cocoa, jam, molasses, syrup, and candies) only contributed sugar (10.0 (1.8) g) and calories (40 (3.5) kcal) to the diet, whereas baked products and pastries (cookies-including whole-wheat cookies, chocolate cookies, cake, croissant, donut, cupcake, batons, or pastries) contributed also lipids (5.9 (0.1) g).Although these foods may provide some micronutrients, their main contribution is usually sugar and saturated fats; therefore they should be consumed only occasionally.

The number of portions of each group and subgroup is proposed in Table 2. Taking into account the level of physical activity, energy and nutritional requirements, three age groups were considered: from 1 to 3 years, from 4 to 10 years, and from 11 to 17 years. In each group different daily caloric needs were considered, depending on the age, sex, and level of physical activity. Daily nutritional intakes were calculated by multiplying the mean nutritional values of each group listed in Table 1 by the number of portions assigned to each age subgroup. The percentages of total energy from fat, protein, and carbohydrates were within the following ranges 30–35:10–15:50–55, respectively. Average protein requirements for children and adolescents (EFSA) as well as intakes of at least 90% of DRI of essential fatty acids, vitamins and minerals were ensured. Different numbers of daily portions for meal planning were considered.

Some aspects of this meal planning deserve consideration: (1) as legumes contribute carbohydrates and protein, each portion of legumes consumed instead of grains could replace one serving of plant protein-rich foods; (2) from 1 to 10 years, it is necessary to select daily two out of these three groups: soy products, plant protein-rich foods, or legumes. If the diet does not include soy products, more legumes or plant protein-rich foods should be consumed instead; (3) higher intake of seeds or nuts than is shown in the table would contribute to the total protein intake: 2 portions a day in children consuming 900–1700 kcal or 4 portions a day in the rest of children could replace one portion of legumes, soy products or plant protein-rich foods; (4) to reach an adequate intake of n-3 fatty acids, a minimum of one portion of walnuts, flax seeds, chia seeds, or flax or walnut oils should be included in all of the patterns proposed; (5) calcium and vitamin D intakes increase notably when fortified plant beverages are chosen. As shown in Table 2, vegan diets do not provide enough vitamin B-12 and vitamin D. Choosing fortified milk substitutes, meat analogues, or breakfast cereals could provide variable amounts of these two vitamins, but supplementation is always recommended. Vitamin D supplementation might not be necessary if sun exposure is sufficient, but this should be evaluated individually. As can be seen in Table 2, with the exception of vitamins D and B12, nutritional needs are easily met with commonly consumed vegan foods, in both low and high caloric dietary patterns (900–3000 kcal/day). 

Finally, applying the previous information regarding food groups, portions, and nutritional content, some dietary recommendations for food selection are listed in Table 3.

## 4. Discussion

We present here the first practical tool specifically designed to facilitate vegan meal planning in children and adolescents. It is based on the same methodology that has been used for the development of exchange lists previously [17,39], and includes commonly foods consumed by vegans.

We have shown that by choosing the proposed number of portions from each subgroup of foods the requirements of key nutrients in vegan diets (protein, calcium, iron, zinc, n-3 fatty acids), are easily met. Requirements of vitamins D and B12 could theoretically be covered as well if fortified foods are included, but because of the wide variability on the level of fortification among these products, it is advisable that these nutrients are provided mainly as supplements [3,6,16]. Another aspect to consider is that some of these fortified products may be high in sugar or salt and therefore not the best option in the long term. Vitamin D supplements might not be necessary in children and adolescents who live in lower latitude areas and spend regular time outdoors.

Although according to the American Academy of Nutrition well-planned vegan diets are adequate for children of all ages, this does not mean that all vegan children are receiving adequate nutrition. This might be partly due to an inappropriate selection of foods caused by incorrect nutritional counselling or by lack of counselling at all. Public nutrition campaigns have been traditionally addressed to families following non-vegetarian diets. Similarly, nutrition education in schools and nutrition training for health professionals have been focused on non-vegan foods and non-vegan meal-planning; the nutritional needs of vegan children have received little attention. We hope that this tool can be used to improve the education of both health professionals and families, which as a result will improve nutritional status in vegan children and adolescents.

As mentioned before, iodine is a nutrient of increasing concern in vegan populations as the iodine content in plant foods is usually low [34]. Because the iodine content of crops differs greatly depending on the geographic region, we have not included this nutrient in our analysis. The World Health Organization recommends salt iodization as the best strategy to prevent iodine deficiency disorders worldwide [41] and therefore vegans should be encouraged to use iodized salt at home. This strategy however has two limitations: (1) whereas in some countries (i.e., Denmark) salt is regularly iodized and its use is mandatory in commercially produced foods like bread [42], other countries like the United Kingdom, still do not iodize their salt [43,44,45]; and (2) many health conscious vegans have reduced or eliminated the use of added salt in their meals. Seaweed may be a valuable source of iodine for vegans, but the iodine content varies between different species and some may have excessive amounts of iodine [46]. Because of the important consequences of iodine deficiency at all ages, but especially in infants and young children, and during pregnancy and breastfeeding, it would be desirable to develop lists of iodine sources as well as recommendations on iodine intakes for vegans. 

### 4.1. Limitations

This study has some limitations. First, we assumed the use of fortified foods to provide calcium requirements, but these products are not always available or chosen by all families. Second, we are aware that vegan diets, as other dietary patterns, includes a great variety of recipes that include variable amounts of macro- and micronutrients. Because they do not fit into one exchange list, developing some guidelines about the number of exchanges per dish should be useful. Also, the practicality and effectiveness of this food exchange system in a clinical setting has not been tested; and this should be done in future studies.

The food exchange system was chosen because we consider food exchange lists very useful to menu planning, since it can be used in individualized dietetic planning or nutrition education. There are other methods for guaranteeing adequate nutrient provision such as linear programming that regulates very precisely the number of calories and macro- and micronutrients in the diet [47]. Nevertheless, it is also important to use methods in which quantities can be easily converted into food portions equivalent to household measures commonly used (spoons, dishes, commercial units), especially in children and adolescents. Also, it is important to be able to choose several foods within a list in order to respect personal preferences (gastronomic and/or cultural). In this sense, as mentioned by other authors the management and adherence to the diet over time is better maintained with tools such as the exchange lists [48].

We have not addressed in this article the specific nutritional requirements of infants younger than 1 year. The main source of nutrients during the first year is breast milk (or formula milk for women who do not wish to breastfeed). The introduction of the first solid foods around 6 months of age and the gradual process of weaning is a critical period in terms of nutrition. Vegan families need and have the same right as the rest of the population to receive high quality professional advice regarding appropriate food choices for their babies and young children.

## 5. Conclusions

These food exchange system specifically developed for children and adolescents following a vegan diet can be used by health professionals to plan balanced vegan meals. They can also be a useful tool for the education of both vegan families and health professionals. 

## Figures and Tables

**Table 1:** Food groups and subgroups with the portion exchanges proposed for the more representative foods of each list expressed in grams and in household measures with the mean energy, macronutrients, and selected micronutrients content per group or subgroup. Data of SD, CV are included.

Food Group	Grams (Householdmeasures)	Values	EnergyKcal	Proteing	CH ^1^g	Fatg	*n*-3g	B12µg	Dµg	Camg	Ironmg	Zincmg
**1. Grains, tubers, and legumes**												
**1.1. Grains and tubers** BreadDry cereals: muesli, cornflakes, oat, bran flakesPastaCereals: rice, millet, spelt, barley, rye, quinoaPotato, sweet potato	60 (2 slices)40 (1/2 bowl)40 (1/2 big portion)40 (1/2 big portion)200 (1 medium)	MeanSDCV	137.39.16.6	4.80.917.6	26.03.012.0	1.20.865.8	0.00.00.0	0.00.00.0	0.00.00.0	15.09.460.7	1.30.752.5	0.90.446.0
**1.2. Legumes** Chickpeas, lentils, beans, adzuki, mungo beans, lupin	30 (2 tbsp)	MeanSDCV	94.18.38.8	6.90.913.6	13.02.217.5	0.70.8113.7	0.00.00.0	0.00.00.0	0.00.00.0	33.711.935.2	2.61.245.1	1.70.318.1
**2. Soy products and fortified plant drinks**												
**2.1. Soy products** Soy drinkSoy yogurtTofu Fermented soy cheese	200 (1 cup)125 (1 container)60 (3 slices)40 (2 slices)	MeanSDCV	78.519.424.8	5.71.221.3	5.54.377.9	3.80.410.8	0.230.33146.0	0.00.00.0	0.00.00.0	136.652.438.5	1.20.973.1	0.71.226.7
**2.2. Fortified plant drinks** Oat, rice, almond drinks	200 (1 cup)	MeanSDCV	94.06.06.4	1.61.275.5	15.73.723.7	2.50.622.3	0.00.00.0	1.50.856.9	1.80.315.7	284.079.728.1	0.90.448.4	0.90.559.2
**3. Plant protein-rich group**												
Textured soy proteinTempehSeitanVegan burger and similar vegan meats	30 (3 tablespoon)60 (3 slices)60 (3 slices)60 (1 piece)	MeanSDCV	98.317.317.6	12.91.914.4	4.12.355.9	3.02.788.1	0.00.00.0	0.71.2173.2	0.00.00.0	60.317.028.2	2.51.454.4	0.90.546.2
**4. Vegetables**												
**4.1. Fresh vegetables** Leafy vegetables, tomato, cucumberPumpkin, carrot, eggplant, artichoke, onions.Sprouted	150 (1 small portion)100 30 (1 handful)	MeanSDCV	28.26.422.7	2.11.047.6	4.31.125.6	0.40.263.0	0.00.00.0	0.00.00.0	0.00.00.0	38.630.779.6	1.00.991.7	0.30.255.1
**4.2. Cruciferous** Cabbages, Brussel sprouts, broccoli, kale	150	MeanSDCV	37.12.67.1	3.40.928.2	4.51.431.5	0.50.19.4	0.00.00.0	0.00.00.0	0.00.00.0	104.627.025.8	1.50.856.5	0.750.228.2
**5. Fruits**												
Fresh fruit: orange, grapefruit, tangerine, nectarine, banana, apple, pear, strawberryDried fruit: raisin, dried plum, dried fig, date, and dried apricot/peach	100–150 (1 piece)30 (1 handful)	MeanSDCV	68.311.016.1	1.10.546.1	15.12.516.8	0.40.280.5	0.00.00.0	0.00.00.0	0.00.00.0	27.70.072.3	1.43.8277	0.30.278.4
**6. Oils, nuts, seeds, and spreads**												
**6.1. Oils and oily fruits** Olive, sunflower, sesame, rapeseed, soy oilsGreen and black olives Avocado, coconut	10 (1 tbsp)40 (1 small portion)40 (half unit)	MeanSDCV	92.89.510.2	1.10.655.8	0.90.880	10.00.959.5	0.30.4126.0	0.00.00.0	0.00.00.0	0.00.00.0	0.00.00.0	0.00.00.0
**6.2. Nuts, seeds and spreads** Sesame, sunflower, pumpkin seeds, almond, hazelnut, pistachio, pine nutTahini, peanut butter and almond butter	20 (1 handful)20 (2 tablespoons)	MeanSDCV	116.914.812.6	3.61.336.6	3.43.294.1	9.92.727.9	0.00.00.0	3.32.371.1	0.00.00.0	41.056.7138.3	1.20.868.7	0.80.454.9
**6.3. *n*-3 fatty acid sources** Flax seeds, chia seeds, walnutsFlaxseed oil	10 (1 tbsp)5 (1 teaspoon)	MeanSDCV	53.78.716.2	1.20.214.6	0.30.394.9	4.71.429.6	1.80.950	1.11.6148	0.00.00.0	24.427.6112.7	0.40.376.9	0.40.4100
**7. Occasional foods**												
**7.1. Baked products** Cookies, croissant, donut, cupcake, pastries	25–30 (units)	MeanSDCV	112.38.97.9	1.70.211.8	13.01.813.8	5.90.711.8	0.00.00.0	0.00.00.0	0.00.00.0	18.06.033.3	0.40.125.0	0.10.00.0
**7.2. Sweeteners** Soluble cocoa, sugar, jam, molasses, syrup	10–15 (1 tbsp)	MeanSDCV	40.03.58.8	0.00.00.0	10.01.818.0	0.00.00.0	0.00.00.0	0.00.00.0	0.00.00.0	9.010.0111.1	0.20.2100.0	0.00.00.0

^1^: Carbohydrates.

**Table 2:** Suggested number of portions of each group and subgroup for vegan children and adolescents considering nutritional requirements and physical activity levels (from sedentary to moderately active ^1^)

	1–3 Years		4–10 Years			11–17 Years			
Food Groups and Subgroups	900 kcal	1200 kcal	1400 kcal	1700 kcal	2000 kcal	2100 kcal	2400 kcal	2700 kcal	3000 kcal
Grains and tubers	3	4	4	5	5	6	6	8	9
Legumes	1	1	1	2	2	2	2	2	2
Soy products	1	1	1	1	1	1	1	2	2
Fortified plant drinks			1	1	1	1	2	2	2
Plant protein-rich group	*	*	*	*	1	1	1	1	2
Fresh and sprouted vegetables	1	1	1	1	2	2	2	2	2
Cruciferous vegetables	1	1	1	1	1	1	1	1	1
Fruits	2	2	3	3	3	3	4	4	5
Vegetable oils	2	2	2	3	3	3	4	4	4
Nuts. seeds and spreads		1	1	1	2	2	2	2	2
*n*-3 fatty acid sources			0.5	0.5	1	1	1	1	1
Energy DRI covered (%)	107	101	100	101	100	102	99	102	101
Protein (g) ^2^	23	30	35	43	50	53	60	68	75
% covered	153	143	133	137	154	156	141	148	158
Carbohydrates (g) ^2^	124	165	193	234	275	289	330	371	413
% covered	109	100	101	100	90	95	92	97	99
Fat (g) ^2^	35	47	54	66	78	82	93	105	117
% covered	85	87	84	87	94	91	94	89	84
n-3 (g) ^3^	0,5	0,7	0,8	0,9	1,1	1,2	1,3	1,5	1,7
Energy DRI covered (%)	107	101	100	101	100	102	99	102	101
Protein (g) ^2^	23	30	35	43	50	53	60	68	75
% covered	153	143	133	137	154	156	141	148	158
Carbohydrates (g) ^2^	124	165	193	234	275	289	330	371	413
% covered	109	100	101	100	90	95	92	97	99
Fat (g) ^2^	35	47	54	66	78	82	93	105	117
% covered	85	87	84	87	94	91	94	89	84
n-3 (g) ^3^	0.5	0.7	0.8	0.9	1.1	1.2	1.3	1.5	1.7
% DRI covered	208	165	260	260	369	354	333	317	329
n-6 (g) ^3^	4.0	5.3	6.2	7.6	8.9	9.3	10.7	12	13.3
% DRI covered	270	264	236	264	271	258	275	245	220
Vitamin B12 (g) ^4.5^	1.5	1.5	1.5	2.5	2.5	3.5	3.5	3.5	3.5
% DRI covered	---	---	102	61	91	65	108	108	129
Calcium (mg) ^4^	450	450	800	800	800	1150	1150	1150	1150
% DRI covered	93	106	100	107	126	89	116	131	140
Iron (mg) ^4^	7	7	11	11	11	12	12	12	12
% DRI covered	183	218	161	196	241	231	250	282	324
Zinc (mg) ^4^	4.3	4.3	5.5	7.4	7.4	10.7	10.7	11	11
% DRI covered	154	193	175	165	196	144	154	173	192

*: Plant protein-rich group at this age is optional and may be alternated with the legumes or the soy products groups. ^1^ Dietary reference intakes for micronutrients. and fatty acids and energy needs calculated for each age group and physical activity level according EFSA DRI report. ^2^ Protein fat and carbohydrate covered considered as 10–15, 30–35, and 50–55 percent of total energy, respectively. ^3^ n-3 and n-6 requirements covered considered as 0.5 and 4 percent of total energy, respectively. ^4^ Vitamins and minerals covered considering DRI in specific age group. ^5^ Vitamin B12 and D requirements covered only if fortified plant drinks or fortified vegan meat alternatives are included. .

**Table 3:** Recommendations for food selection with food groups and subgroups.

Food Groups and Subgroups	Practical Recommendations
1. Grains and derivatives, tubers, and legumes	- Whole grain cereals should be chosen over refined grains.Some breakfast cereals are fortified with vitamin D and vitamin B-12.Soaking and sprouting grains and legumes before cooking them may improve iron and zinc bioavailability; a similar effect may occur with sour leavening (sourdough bread).
2. Soy products and fortified plant drinks	Soy products count as half portion of the plant protein-rich group. If soy products are not consumed, the intake of legumes and/or plant protein-rich products should be increased. It is advisable to select calcium-fortified plant drinks; it is desirable but not essential if they are also fortified with vitamins D and B12.
3. Plant protein-rich group	Although some foods of this group are fortified with vitamin B-12, daily or weekly supplementation according to guidelines is recommended.It is advisable to select vegan burgers, sausages, and other vegan meats that are made using unsaturated fats and that have a low salt content.
4. Vegetables: cruciferous and other vegetables	If fortified plant drinks are not consumed, it is advisable to increase the number of portions of cruciferous vegetables (as well as calcium-rich nuts and seeds—chia, sesame, almonds) and calcium-set tofu.It is advisable to include carotenoid-rich vegetables regularly (sweet potato, carrot, pepper, spinach, pumpkin) as they are the main source of vitamin A in vegan diets.
5. Fruits: fresh and dried	Dried fruits are especially valuable in children and adolescents with higher caloric requirements, and as they are also a good source of vitamins and minerals are preferable to baked products or sweets.
6. Oils, nuts, seeds and spreads	Olive and rapeseed oils should be chosen over sunflower, soy and other n-6 rich vegetable oils. At least half to one portion in this group should be rich in n-3 fatty acids.Two portions of seeds or nuts count as one portion of legumes in terms of protein content.If plant drinks are not calcium-fortified, it is advisable to include one extra portion of sesame seeds or sesame paste (tahini).
7. Occasional foods:Sugars and baked products	Sweets and baked products are not essential and should be consumed in moderation (not more than one portion per day). Baked products and pastries would count as one portion of the grain group plus one portion of oils, seeds, nuts and spreads; however, their nutritional value in terms of micronutrients is much poorer.If plant drinks are sweetened they count as a portion of sugar.
